# La Crosse virus infectivity, pathogenesis, and immunogenicity in mice and monkeys

**DOI:** 10.1186/1743-422X-5-25

**Published:** 2008-02-11

**Authors:** Richard S Bennett, Christina M Cress, Jerrold M Ward, Cai-Yen Firestone, Brian R Murphy, Stephen S Whitehead

**Affiliations:** 1 Laboratory of Infectious Diseases, National Institute of Allergy and Infectious Diseases, National Institutes of Health, Bethesda, MD 20892, USA; 2 Infectious Disease Pathogenesis Section, Comparative Medicine Branch, Division of Intramural Research, National Institute of Allergy and Infectious Diseases, National Institutes of Health, Bethesda, MD 20892, USA

## Abstract

**Background:**

La Crosse virus (LACV), family Bunyaviridae, was first identified as a human pathogen in 1960 after its isolation from a 4 year-old girl with fatal encephalitis in La Crosse, Wisconsin. LACV is a major cause of pediatric encephalitis in North America and infects up to 300,000 persons each year of which 70–130 result in severe disease of the central nervous system (CNS). As an initial step in the establishment of useful animal models to support vaccine development, we examined LACV infectivity, pathogenesis, and immunogenicity in both weanling mice and rhesus monkeys.

**Results:**

Following intraperitoneal inoculation of mice, LACV replicated in various organs before reaching the CNS where it replicates to high titer causing death from neurological disease. The peripheral site where LACV replicates to highest titer is the nasal turbinates, and, presumably, LACV can enter the CNS via the olfactory neurons from nasal olfactory epithelium. The mouse infectious dose_50 _and lethal dose_50 _was similar for LACV administered either intranasally or intraperitoneally. LACV was highly infectious for rhesus monkeys and infected 100% of the animals at 10 PFU. However, the infection was asymptomatic, and the monkeys developed a strong neutralizing antibody response.

**Conclusion:**

In mice, LACV likely gains access to the CNS via the blood stream or via olfactory neurons. The ability to efficiently infect mice intranasally raises the possibility that LACV might use this route to infect its natural hosts. Rhesus monkeys are susceptible to LACV infection and develop strong neutralizing antibody responses after inoculation with as little as 10 PFU. Mice and rhesus monkeys are useful animal models for LACV vaccine immunologic testing although the rhesus monkey model is not optimal.

## Background

La Crosse virus (LACV), family *Bunyaviridae*, is a mosquito-borne pathogen endemic in the United States [1,2]. The LACV genome consists of three single-stranded, negative-sense RNA genome segments designated small (S), medium (M), and large (L). The S segment encodes two proteins in overlapping reading frames: the nucleoprotein (N) and a non-structural protein (NS_S_) which suppresses type 1 interferon (IFN) in the mammal host. The M segment encodes a single polyprotein (M polyprotein) that is post-translationally processed into two glycoproteins (G_N _and G_C_), and a non-structural protein (NS_M_) [3]. G_N _and G_C _are the major proteins to which neutralizing antibodies are directed [4,5]. The L segment encodes a single open reading frame for the RNA-dependent RNA polymerase (L) [6].

The virus is transmitted by hardwood forest dwelling mosquitoes, *Aedes triseriatus*, which breed in tree holes and outdoor containers. *Ae. triseriatus *feed on Eastern chipmunks (*Tamias striatus grinseus*) and Eastern gray squirrels (*Sciurus carolinensis*) which serve as amplifying hosts for LACV [7-9]. Interestingly, the virus can be maintained in the mosquito population in the absence of vertebrate hosts by transovarial (vertical) transmission, thus allowing the virus to over-winter in mosquito eggs [9]. More recently, LACV has been isolated from naturally infected, non-native *Aedes albopictus *mosquito [10]. The infection of *Ae. albopictus *may represent a shift in virus ecology and increases the possibility for generation of new reassortants [11].

LACV was first identified as a human pathogen in 1960 after its isolation from a 4 year-old girl from Minnesota who suffered meningoencephalitis and later died in La Crosse, Wisconsin[12]. In humans, the majority of infections are mild and attributed to the "flu" or "summer cold" with an estimated 300,000 infections annually, of which there are only 70–130 serious cases reported [1,2,13,14]. Isolation of virus is rare and has been made from post-mortem brain tissue collected in 1960, 1978, and 1993 [15-18]. Two isolates from non-fatal LACV cases were collected in 1995, one from a brain biopsy of a child and one from cerebrospinal fluid [16,19].

Histopathologic changes in two human cases, one from 1960 and one from 1978, were characteristic of viral encephalitis. Inflammatory lesions consisted of infiltration of mononuclear leukocytes either diffusely or as microglial nodules. The largest inflammatory foci were observed in the cerebral cortex, including the frontal, parietal, and temporal lobes, and foci could also be found in the basal ganglion and pons. In these two cases, there was a lack of inflammatory lesions in the posterior occipital cortex, cerebral white matter, cerebellum, medulla, and spinal cord [17]. Brain biopsy from a non-fatal LACV infection contained areas of perivascular mononuclear cuffing and focal aggregates of mononuclear and microglia cells [16]. RT-PCR analysis of neural tissues from the 1978 case could only detect viral RNA in the cerebral cortex and not in the medulla, cerebellum, spinal cord, basal ganglion, or temporal lobe [20].

In children and adults, severe LACV encephalitis clinically mimics herpes simplex virus encephalitis with fever, focal signs, and possible progression to coma [16,21,22]. Confirmatory diagnosis has been made by RT-PCR of cerebrospinal fluid to exclude herpes simplex virus and by fluorescent staining for LACV antigen in brain biopsy sections [16]. Children who recover from severe La Crosse encephalitis may have significantly lower IQ scores than expected and a high prevalence (60% of those tested) of attention-deficit-hyperactivity disorder [13]. Seizure disorders are also common in survivors [23]. Projected lifelong economic costs associated with neurologic sequelae range from $48,775 – 3,090,398 per case [24]. Currently, a vaccine or specific antiviral treatment is not available, but could serve to reduce the clinical and economic impact of this common infection.

Although evidence of LACV infection has been reported for several species, only limited research has been done to understand LACV pathogenesis in its natural host or experimental rodents [8,25-27]. LACV administered subcutaneously to suckling mice first replicates in muscle, and viremia develops with virus invading the brain across vascular endothelial cells [28-31]. Virus replication in muscle was confirmed by immunohistochemical (IHC) staining, and the predominant cell type infected in the CNS is the neuron [28,32]. The virulence of LACV for mice decreases with increasing age, similar to humans in which it causes CNS disease predominantly in pediatric populations [13,28]. As an initial step in the establishment of animal models useful for vaccine development for humans, we sought to better characterize the tissue tropism of LACV in mice by identifying the tissues that support LACV replication after peripheral inoculation. We have previously described Swiss Webster mice as suitable for characterization of LACV infection at birth and at 3-weeks of age [6]. Here we inoculated 3-week old Swiss Webster mice with either 1 or 100 LD_50 _of virus intraperitoneally. Twenty tissues were individually collected for six consecutive days and processed for virus titration, immunohistochemical staining, and histopathology studies.

Since experimental infection of non-human primates with LACV has not been reported, we also sought to determine if rhesus monkeys were susceptible to LACV infection. Rhesus monkeys were chosen since they are susceptible to a variety of neurotropic arboviruses, including flaviviruses [33]. In this study, rhesus monkeys were infected intramuscularly or subcutaneously with a mosquito or human isolate of LACV. These two isolates were used since preliminary genomic sequence analysis indicate that there are host specific differences between LACV isolated from humans and mosquitos [6]. LACV was found to be highly infectious for rhesus monkeys, but infection did not result in viremia, disease, or significant changes in blood chemistries or cell counts. However, high titers of neutralizing antibodies developed in all monkeys indicating that rhesus monkeys, although not optimal, will be useful for studying the infectivity and immunogenicity of LACV vaccine candidates.

## Results

### LACV replicates in various tissues after intraperitoneal inoculation of mice

**Figure 1:**
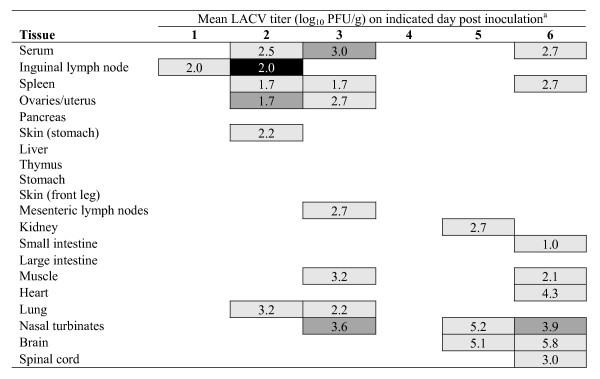
**Tissue distribution of La Crosse virus following intraperitoneal (IP) inoculation of Swiss Webster mice with 10^2.5 ^PFU**. ^a^Percent of mice positive by plaque assay represented by shading: 100% black, 66% dark gray, 33% light gray, 0% no data entry. Mean virus titer calculated only for virus positive tissues. Areas left blank indicate virus titer below detection limit of 0.7 log10 PFU/tissue.

**Figure 2:**
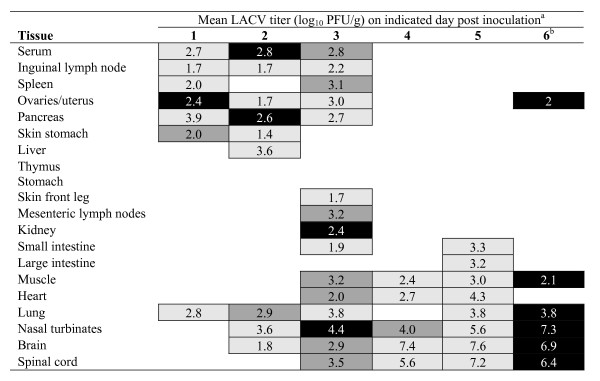
**Tissue distribution of La Crosse virus following intraperitoneal (IP) inoculation of Swiss Webster mice with 10^4.5 ^PFU**. ^a^Percent of mice positive by plaque assay represented by shading: 100% black, 66% dark gray, 33% light gray, 0% no data entry. Mean virus titer calculated only for virus positive tissues. Areas left blank indicate virus titer below detection limit of 0.7 log_10 _PFU/tissue. ^b^Tissue samples collected from one moribund mouse.

Previous evaluation of LACV in suckling mice revealed that it first replicated in striated muscle cells from which it seeded the blood and next invaded the CNS, where it replicated in neurons [28,32]. In developing a rodent model for our live attenuated LACV vaccine development program, we sought to characterize LACV infection in outbred weanling mice, rather than suckling mice, since older mice can be used to study both the level of attenuation and the immunogenicity of our LACV vaccine candidates. To identify key steps in pathogenesis of LACV in weanling mice, we evaluated LACV tissue tropism after peripheral inoculation of wild-type virus and sought to identify tissues in which virus replicated efficiently. Weanling Swiss Webster mice (21–23 days-old) were inoculated intraperitoneally with 1 or 100 LD_50 _(2.5 or 4.5 log_10 _PFU) of LACV/human/1960, and the tissues indicated in Figures 1 and 2 were collected from 3 mice per day on days 1–6 post inoculation. Following inoculation of either dose, virus could first be detected in tissue near the inoculation site such as inguinal lymph nodes, spleen, and ovaries/uterus. Virus was detected in serum on days 1–3, but rarely on subsequent days even in moribund mice. By day three, virus distribution was widespread and could be found in the majority of tissues sampled, albeit at very low levels with titers rarely exceeding those found in serum. The highest virus titers detected were present in nasal turbinates, brain, and spinal cord. Respiratory tissue, including lung and nasal turbinates, contained virus following inoculation with 100 or 1 LD_50 _beginning on days 1 and 2, respectively. CNS infection appeared to follow respiratory tissue infection, with virus being present in the brain 5 days after infection with 1 LD_50 _and on day 2 after infection with 100 LD_50_. At either virus dose, infection appears early in the lymph nodes and major organs, with subsequent infection of the upper respiratory tract (nasal turbinates) followed by infection of the brain and eventually the spinal cord. By day six, mice began to succumb to infection in the high dose group showing signs of paralysis whereas mice in the low dose group failed to show clinical signs at this time, but would have succumbed later in infection. These results indicate that LACV replicates to low to moderate levels in peripheral tissues in weanling mice, with the nasal turbinates rather than striated muscle being the major site of replication.

### Intranasal infection of mice with LACV

**Figure 3:**
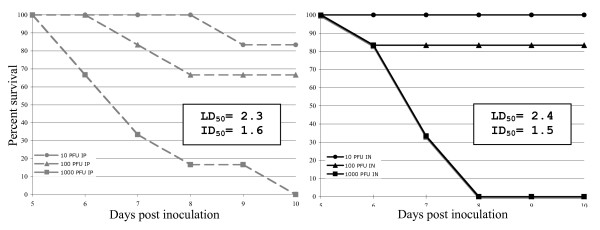
**LACV is highly virulent in mice inoculated by either the intraperitoneal (IP) or intranasal (IN) route**. Percent survival for LACV/human/1960 after IP (left) or IN (right) inoculation routes. Changes in percent survival did not occur after day 10.

Since LACV replicated to high titers in the nasal turbinates, we sought to determine if intranasal inoculation of mice with LACV could lead to infection. Three-week-old Swiss Webster weanling mice (n = 6/dose) were inoculated intranasally (IN) (10 μl volume) or intraperitoneally (IP) (100 μl volume) with serial dilutions of LACV/human/1960, and the LD_50 _and 50% infectious dose (ID_50_) were determined. Clinical disease served as a surrogate for lethality and mice were promptly euthanized prior to succumbing to LACV disease. In both groups, clinical disease was first noted on day 6 (Figure 3). Twenty days post-inoculation, the LD_50 _was determined. All surviving mice were tested for the development of a neutralizing antibody response. To determine the ID_50 _titer, mice were considered infected if they either developed clinical disease or a serum neutralizing antibody titer. The LD_50 _was similar in both the IN and IP groups (2.4 and 2.3 log_10 _PFU, respectively) with the LD_50 _following IP injection in agreement with previous experiments [6]. The ID_50 _titers (1.5 and 1.6 log_10 _PFU for the IN and IP routes, respectively) were slightly lower than the LD_50 _titers, indicating LACV can cause a subclincal infection in weanling mice, but only at low doses.

### Histopathology and Immunopathology in mice infected with LACV 100 LD_50_

**Figure 4:**
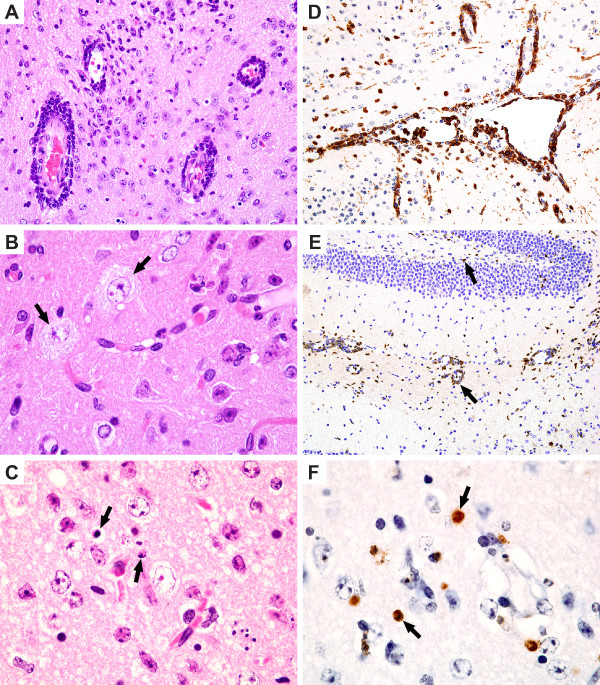
**Histopathologic changes on day 6 in the CNS of LACV infected mice. (A) **Perivascular cuffs and gliosis in the thalamus. H&E X400. (**B) **Neurons of the cervical spinal cord with degenerative changes of pale cytoplasm and vacuoles (arrows). H&E X1000. **(C) **Thalamus with apoptotic cells (arrows) and degenerative neurons (with swollen vacuolated nuclei). H&E X1000. **(D) **CD3+ lymphocytes in the meninges and blood vessels in the thalamus. Immunohistochemistry, hematoxylin counterstain, X200. **(E) **Macrophages (MAC-2 +) in perivascular cuffs and areas of gliosis in the hippocampus. Immunohistochemistry, hematoxylin counterstain X200.**(F) **TUNEL positive (brown) apoptotic bodies in the thalamus. Immunohistochemistry, hematoxylin counterstain X1000.

**Figure 5:**
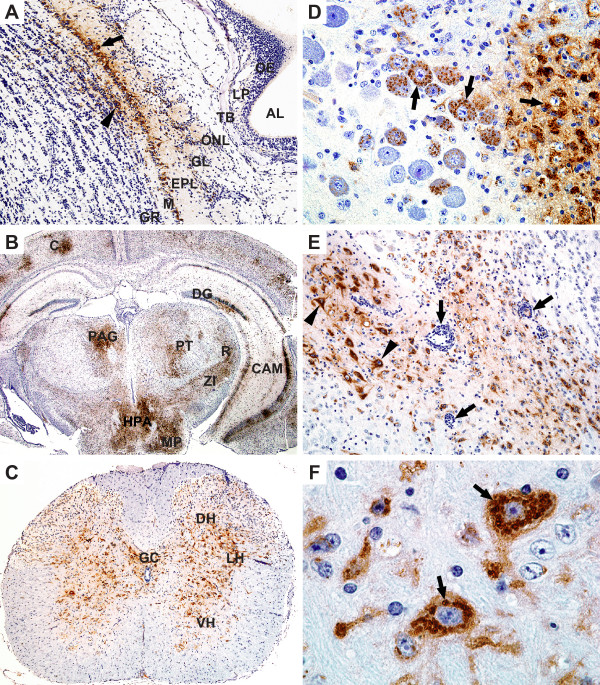
**Viral antigen is detected in the CNS of LACV infected mice. (A) **LACV antigen-positive cells in the mitral cell layer (arrow) and granule cell layer (arrowhead) of the main olfactory bulb. Abbreviations: AL-airway lumen, OE-olfactory epithelium, LP-lamina propria, TB-turbinate bone, ONL-olfactory nerve layer, GL-glomerular layer, EPL- external plexiform layer, M-mitral cell layer, GR-granule cell layer. Day 6, X100. **(B) **Low magnification of a coronal section of mouse brain showing abundant La Crosse viral antigen. Abbreviations: C-cerebral cortex, CAM-cornu ammonis of hippocampus, DG-dentate gyrus, HPA-posterior hypothalamic area, MP-premamillary nucleus, PAG-periaqueductal gray, PT-pretectum, R-reticular nucleus of thalamus, ZI- zona inserta. Day 6, X12.5. **(C) **Cervical spinal cord cross section showing abundant La Crosse viral antigen in grey matter. Abbreviations: GC-gray commissure, DH-dorsal horn, LH-lateral horn, VH-ventral horn. Day 6, X100. **(D) **Viral antigen in a punctate pattern in neurons (arrows) in the locus coeruleus. Day 5, X400. **(E) **Medulla oblongata with abundant LACV antigen in many neurons (arrow heads) with associated perivascular lymphocyte cuffing (indicated with arrows). Day 4, X200. **(F) **La Crosse viral antigen in the cytoplasm of medullary neurons (arrows). Day 6, X1000. All images immunohistochemistry, hematoxylin counterstain.

To further characterize the LACV infection in weanling mice, an additional group was inoculated intraperitoneally with 100 LD_50 _of LACV/human/1960 and selected tissues (serum, muscle, nasal turbinate, brain, and spinal cord) were collected for virus quantitation (n = 5, daily for six days) to confirm titers found in Figure 2 and for histopathological and immunohistochemical (IHC) examination (n = 3, daily for six days), and the data is summarized in Table 1 and Figures 4 and 5. Virus titers for nasal turbinates, brain, spinal cord, muscle, and serum were in agreement with findings in Figure 2 (data not shown). Histopathologic lesions in the brain (including areas of the olfactory bulb, cerebral cortex, thalamus, hippocampus, and medulla oblongata) and spinal cord included perivascular cuffing (Figure 4A), neuronal degeneration (Figure 4B–C), necrosis (either single cell or small foci), and apoptosis (4F). There was an infiltration of CD3+ lymphocytes and macrophages (Figure 4D–E). The most extensive lesions occurred in the medulla oblongata and were associated with perivascular cuffing.

**Table 1:** Spread of virus from brain to spinal cord after IP inoculation with 10^4.5 ^PFU LACV/human/1960.

	No. of mice with detectable viral antigen or histopathologic lesions^a^															
	
	Brain										Spinal cord					
		
	Olfactory bulb		Thalamus		Cerebral cortex		Medulla		Cerebellum		Cervical		Thoracic		Lumbar	
	
Day	IHC^b^	HE^c^	IHC	HE	IHC	HE	IHC	HE	IHC	HE	IHC	HE	IHC	HE	IHC	HE
1	0	0	0	0	0	0	0	0	0	0	0	0	0	0	0	0
2	0	0	0	0	0	0	0	0	0	0	0	0	0	0	0	0
3	0	0	0	0	0	0	0	0	0	0	0	0	0	0	0	0
4	1	1	1	1	1	0	1	1	0	0	0	0	0	0	0	0
5	3	0	3	1	2	0	1	0	1	0	1	1	1	1	1	1
6	3	2	3	3	3	2	3	2	1	0	2	2	2	1	1	0

^a ^Data displayed as total positive by IHC and HE (n = 3). Viral antigen or lesions were not observed in nasal turbinates, muscle, spleen, or pancreas. Tissues from three control mice were collected on days 1 and 6 and processed for HE and IHC comparisons, viral antigen or lesions were not present.

^b ^Immunohistochemical stain

^c ^Hemotoxylin and eosin stain

Histopathological changes were minimal outside the CNS. In the spleen, lymphoid atrophy was only observed on days 3–4 post-infection. Plasmacytosis in the spleen was observed on days 4–6 and areas of necrosis were observed on days 4–5 with myeloid hyperplasia on day 5. Histopathological changes were not observed in respiratory nasal epithelium or muscles of the upper rear limb.

To determine the location of cells expressing viral antigen, tissues were immunostained for La Crosse virus antigens. Viral antigens were not observed in the nasal turbinates, muscle, spleen, or pancreas. However, viral antigen was detected in the olfactory bulb of the brain, thalamus, cerebrum, medulla oblongata, and spinal cord.

On day 5, viral antigen could be detected in all sampled brain regions with a greater number of cells and regions positive for viral antigen than for overt histologic lesions. Mild perivascular cuffing was seen in a few areas and single cell necrosis was seen in areas stained positive for viral antigen. At this time and at later days, the olfactory bulb was more extensively involved (Figure 5A), although viral antigen was not detected in sections of the underlying nasal epithelium. Viral antigen could be detected in the brain tissues of all mice on day 5, but only small foci of antigen were seen in the spinal cord of one mouse suggesting brain infection proceeds spinal cord infection.

By day 6, viral infection in the CNS was widespread and extensive throughout all regions of the brain and spinal cord (Figure 5B). Neurons were the main cell expressing viral antigens, but supporting glia also appeared positive (Figures 5D–F). Single cell necrosis, focal necrosis (more than one cell), and apoptotic bodies were prominent throughout the lesions (Figure 4C) and apoptotic bodies stained positive by TUNEL staining (Figure 4F). Degenerative neuronal changes were also commonly seen, including nuclear vacuolization and cell shrinkage (Figure 4B–C). To identify cell infiltrates in a moribund mouse, brain sections were immunostained using anti-CD3 or anti-macrophage antigen-2 (MAC-2) antibodies. CD3+ cells and macrophages were seen in perivascular locations and in areas of the neuronal lesions (Figure 4D–E). Within the grey matter of the spinal cord, there were small perivascular cuffs of lymphocytes and degenerative neurons with viral antigen expressed in numerous neurons (Figure 4B, 5C). On day 6, 2 of 3 of the mice had abundant viral antigen in the cervical and thoracic regions of the spinal cord, and only one mouse had viral antigen in the lumbar spinal cord supporting the observation the infection travels caudally from the brain down the spinal cord.

### Inoculation of rhesus monkeys with LACV

To develop a non-human primate model of LACV infection for pathogenesis studies and for testing of future vaccine candidates, rhesus monkeys were inoculated with 10^5 ^PFU of biologically cloned (terminally diluted) human or mosquito LACV (LACV/human/1978-clone, LACV/mosquito/1978-clone). Illness was not observed following virus administration, and virus was not detected at any time in serum samples (Table 2). Virus was present at a low titer in lymph nodes on days 6, 8, and 12, however, virus replication in these tissues could not be identified by IHC staining. Despite the low level (or absence) of viremias and highly restricted replication in the tissues sampled, all monkeys developed neutralizing antibody responses that were first detected on days 6–8 indicating that the each monkey was infected. Twenty-eight days after inoculation, neutralizing antibody titers (PRNT_60_) for each group were in the range of 1:560 – 1:2186 (Table 2). Low-level cross-reactive antibodies were present in two monkeys (CL6E and DBOH) at the start of the experiment. A boost in antibody titer in these monkeys was not observed compared to other monkeys, suggesting that this experimental LACV infection was a primary infection.

**Table 2:** LACV is immunogenic in rhesus monkeys and can be detected in the lymph nodes.

				Homologous neutralizing antibody titer on indicated day (reciprocal PRNT_60_)^c^											
					
Virus and inoculation route	Monkey #	Temp. (°F)^a^	Lymph node virus titers^b^	-7	0	2	4	6	8	10	12	14	21	28	GMT^d ^(Day 28)
Human/1978/clone (SQ)	DB00		2.3 (day 12)	<5	<5	<5	<5	<5	13	22	166	620	1465	2924	2186
	DB1Z			<5	<5	<5	<5	<5	24	137	126	332	679	1412	
	CL74			<5	<5	<5	<5	<5	<5	31	199	546	1370	1374	
	DBCV			<5	<5	<5	<5	<5	6	43	246	234	1684	4022	

Human/1978/clone (IM)	DB9J			<5	<5	<5	<5	<5	<5	66	343	1011	603	1776	1906
	DB5C		1.4 (day 12)	<5	<5	<5	<5	<5	<5	74	282	2382	7451	2714	
	DB7Y	103.2 (day 8)		<5	<5	<5	<5	<5	<5	15	37	230	4041	1628	
	DB3W	102.8 (day 6)		<5	<5	<5	<5	<5	<5	40	1643	460	2633	1688	

Mosquito/1978/clone (SQ)	DB70			<5	<5	<5	<5	37	25	90	623	672	2325	3008	560
	DB8N		0.7 (day 12)	<5	<5	<5	<5	12	29	72	194	161	211	663	
	CK74			<5	<5	<5	<5	<5	14	38	250	434	1023	399	
	CL2G			<5	<5	<5	<5	7	7	8	19	14	120	124	

Mosquito/1978/clone (IM)	DBCZ			<5	<5	<5	<5	12	38	834	1325	3050	3695	4493	1691
	CL6E	103.1 (day 6)	0.7 (day 6)	5	6	6	<5	16	39	180	1960	2809	3875	960	
	DB0H			13	13	18	13	15	28	130	516	1566	1134	687	
	DA9F	102.9 (day -7)	0.7 (day 8)	<5	<5	<5	<5	<5	11	94	1833	3189	3656	2762	

^a ^Rectal temperature collected at time of blood draw. Temperatures outside the normal range (98–102.7 F) indicated by day in parenthesis, normal values left blank.

^b ^Lymph nodes were collected on days 4, 6, 8, and 12 for virus titer and IHC staining. Viral titers are expressed as log_10 _PFU/half lymph node (day post inoculation). Viral antigen was not observed using IHC staining. Blank spaces indicate that virus was not detected in the lymph nodes. Lower limit of detection was .7 log_10 _PFU/tissue.

^c ^Limit of detection for neutralizing antibodies is 1:5 dilution.

^d ^Geometric mean neutralizing antibody titer.

Complete blood counts (CBC) with differential and blood chemistries were analyzed at each blood collection. Since LACV infection in monkeys was asymptomatic and also since differences between the four virus groups indicated in Table 2 were not observed, the CBC and blood chemistry data were averaged for the 16 animals to detect changes in blood values during the course of infection (Table 3). Days in which specific parameter values for a significant number of monkeys were greater than 1 standard deviation from normal appear boxed in Table 3 with mean values for each test shown. After day 6, the majority of monkeys experienced a slight anemia, which may in part be associated with the overnight fast in preparation for anesthesia prior to each blood collection. This analysis suggests that infection of major organs such as liver was minimal or absent.

**Table 3:** Infection of rhesus monkeys with LACV results in limited changes in blood chemistries or cell counts.

			**Mean test value on indicated day post inoculation**^a^								
			
**Test**^b^	**Unit**	**Mean ± SD**^c^	2	4	6	8	10	12	14	21	28
**CBC**											
White blood count	THSN/UL	9.7 ± 2.9	7.9	9.2	9.2	8.3	8.2	8.5	8.0	6.7	7.2
Red blood count	MILL/UL	5.7 ± 0.3	5.5	5.4	5.3	**4.8**	**5.0**	5.2	**5.2**	5.4	**5.1**
Hemoglobin	GM/DL	12.8 ± 0.5	12.7	12.3	**11.9**	**11.0**	**11.5**	**11.6**	**11.4**	**12.1**	**11.8**
Hematocrit	Percent	38.9 ± 1.7	38.7	37.7	36.8	**33.8**	35.8	**35.8**	**35.8**	38.2	37.1
MCV	FL	68.5 ± 2.7	71	70.4	68.9	68.1	71.1	68.6	69.9	70.2	72.2
MCH	PICO GM	22.5 ± 0.8	23.3	23.0	22.4	22.4	22.8	22.2	22.0	22.2	23.0
MCHC	Percent	32.9 ± 0.8	32.9	32.7	32.5	32.7	32.0	32.4	**31.4**	**31.7**	**31.9**
Platelet	THSN/UL	391.7 ± 83.6	419.4	371.3	348.2	395.3	449.9	432.2	431.4	445.2	389.5

**Differential**											
Absolute polys	THSN/UL	3550.7 ± 1779.4	2057.5	4299.9	3888.3	4243.2	3539.1	3649.1	2758.6	2291.6	2510.0
Bands	THSN/UL	0 ± 0	0	0	0	0	0	0	0	0	0
Lymphocytes	THSN/UL	5301.9 ± 1830.4	5142.9	4347.6	4773.3	3727.5	4054.3	4300.8	4187.0	3817.5	3803.4
Monocytes	THSN/UL	342.9 ± 148.0	362.1	351.6	365.4	297.1	341.0	277.2	272.0	253.8	241.5
Eosinophils	THSN/UL	257.6 ± 181.1	209.8	200.8	230.0	167.9	290.6	235.9	213.7	229.6	180.8
Basophiles	THSN/UL	0 ± 0	0	0	0	8.1	0	0	0	13.8	0

**Chemistry**											
Sodium	MEQ/L	152.2 ± 3.5	152.2	152.2	152.2	152.2	152.2	152.2	152.2	152.2	152.2
Potassium	MEQ/L	3.9 ± 0.5	4.2	3.9	3.8	3.6	4.1	3.8	4.1	4.2	3.8
Chloride	MEQ/L	110.6 ± 3.5	110.6	110.2	109.1	108.1	110.4	105.0	107.1	110.3	106.7
Calcium total	MG/DL	9.7 ± 0.3	9.5	9.5	**9.3**	**9.0**	9.4	9.6	9.4	9.6	**9.8**
Phosphorus	MG/DL	5.9 ± 1.0	5.9	5.8	5.4	5.6	5.9	5.8	5.6	5.8	6.7
Magnesium	MEQ/L	1.7 ± 0.2	1.8	1.7	1.6	1.7	1.8	1.6	1.6	1.6	1.8
AST (SGOT)	U/L	38.5 ± 7.4	39.1	43.7	46.8	41.3	43.2	48.4	39.6	36.4	55.0
ALT (SGPT)	U/L	29.4 ± 9.5	31.2	34.1	35.9	36.4	34.8	34.8	33.0	26.5	30.9
ALP	U/L	692.0 ± 166.2	604.5	586.1	597.0	597.8	510.1	532.6	548.5	529.4	553.5
Amylase	U/L	203.0 ± 56.0	231.5	219.3	211.1	203.2	221.8	228.8	216.5	206.3	215.6
Glucose	MG/DL	65.0 ± 11.3	76.9	88.6	116.7	132.8	70.4	66.8	65.4	64.8	66.8
BUN	MG/DL	20.0 ± 7.5	18.5	19.2	16.6	18.1	22.9	18.9	18.7	16.3	22.0
Creatinine	MG/DL	0.8 ± 0.2	0.7	0.8	0.7	0.8	0.7	0.7	1.2	0.8	0.8
Cholesterol	MG/DL	153.8 ± 31.4	157.1	153.6	139.8	142.3	158.6	150.3	148.6	146.3	151.6
Triglyceride	MG/DL	65.3 ± 21.7	47.1	59.9	75.4	49.5	37.7	61.1	70.5	58.7	60.8
Bilirubin, total	MG/DL	0.2 ± 0.1	0.2	0.2	0.2	0.2	0.2	0.2	0.2	0.1	0.2
Albumin	G/DL	4.5 ± 0.2	4.5	4.5	4.2	**4.2**	4.4	4.3	**4.2**	4.3	4.6
Protein, total	G/DL	6.9 ± 0.3	6.9	6.9	6.5	6.5	6.9	6.6	6.6	6.8	7.2
Globulin	G/DL	2.4 ± 0.2	2.4	2.4	2.3	2.4	2.5	2.4	2.4	2.6	2.6
Lipase-PS	U/L	32.4 ± 27.6	30.4	27.7	53.1	25.4	32.2	38.4	39.5	33.1	28.7
Osmolality, Calculated	mOsm/kg	306.0 ± 55.1	298.1	300.1	299.6	292.6	297.1	286.8	288.1	296.3	288.9

^a^Days with significant number of monkeys 1 SD from the pre-inoculation mean (day -7 and 0 combined) are boxed (chi square, p < 0.05, n = 16), mean values displayed.

^b^Abbreviations: MCV-mean corpuscular volume, MCH-mean corpuscular hemoglobin, MCHC-mean corpuscular hemoglobin concentrations, AST-aspartate aminotransferase, ALT-alanine aminotransferase, ALP-alkaline phosphatase, BUN-blood urea nitrogen.

^c^Pre-inoculation mean determined from samples collected on days -7 and 0 post inoculation.

To estimate the minimum dose required to infect a monkey, rhesus monkeys were inoculated with LACV/mosquito/1978-cl at 10^1 ^or 10^3 ^PFU subcutaneously. Blood was collected on days 0, 21, 28, and 42, and serum neutralizing antibody titers were determined. Mean neutralizing antibody titers were 1:355 and 1:82 for the 10^1 ^or 10^3 ^PFU dose groups, respectively, and all monkeys seroconverted by day 28 (PRNT_60 _≥ 40) (Table 4). Thus, LACV is highly infectious for rhesus monkeys even at a dose of 10^1 ^PFU and results in the induction of a high level of neutralizing antibody. However, LACV infection did not result in identifiable clinical abnormalities in this group of 24 monkeys.

**Table 4:** LACV/mosquito/1978 is highly infectious in rhesus monkeys following subcutaneous inoculation.

		Homologous neutralizing antibody titer on indicated day (reciprocal PRNT_60_)^a^			
		
Dose PFU (SQ)	Monkey #	0	21	28	42
10^3^	DB5C	<10	1131	156	41
10^3^	DBNF	<10	1236	420	230
10^3^	DBFD	<10	151	157	128
10^3^	DBNL	<10	25	49	37
		**GMT: <10**	**270**	**150**	**82**

10^1^	DBPJ	<10	1175	783	300
10^1^	DBKL	<10	1529	831	755
10^1^	DBTB	<10	150	282	503
10^1^	DBKA	<10	2616	448	140
		**GMT: <10**	**916**	**535**	**355**

^a^Limit of detection for neutralizing antibodies is 1:10 dilution

## Discussion

As an initial step in development of a live attenuated LACV vaccine, we sought to better characterize LACV infection in weanling mice because at this age mice can be used to evaluate both the level of attenuation and immunogenicity of candidate vaccine viruses. Previous LACV pathogenesis studies in suckling mice inoculated subcutaneously with a mosquito isolate of LACV demonstrated that viral antigen was detected in the cytoplasm of striated muscles, the interscapular brown fat, and the endothelial and smooth muscle cells of small arteries and veins [28]. When virus was first detected in the brain, it was confined to cerebral vascular endothelial cells but later spread to neurons. The authors suggest that the late infection of the dorsal route ganglion indicates that the virus does not penetrate the CNS via nerves but rather by vascular endothelial cells [28]. This previous model therefore suggests that virus first replicates in muscle cells leading to the development of viremia with subsequent hematogenous spread to the CNS, and we sought in the present study to examine if this pattern of infection also occurred in weanling mice.

In weanling mice inoculated intraperitoneally with 1 or 100 LD_50 _of LACV, virus was first detected on days 1 – 3 in tissues near the inoculation site. At either dose, virus was no longer detectable by days 4 and 5 in these tissues, suggesting that it was rapidly cleared by the innate immune system. Interestingly, the virus was not detected in muscle tissue until day 3 post inoculation and clearly did not preferentially infect this tissue. Rather, outside the CNS, the virus replicated to highest titers in the nasal turbinates and appears to spread from this site into the brain. Immunohistochemical staining of the nasal turbinate tissue was not sensitive enough to identify the LACV infected cells, but is thought LACV may gain access to the CNS via cells in the nasal turbinates. This suggestion is offered with the caveat that respiratory epithelial cells could also have been infected, but the magnitude of the infection could not be detected with the IHC staining. To travel from the nasal olfactory epithelium to the olfactory bulb, the virus would follow olfactory neurons into the brain, as infection is first detected in the rostral section of the brain.

Although virus replication in nasal turbinate tissue was detected, we were unable to identify the cells that were infected. Viruses such as vesicular stomatitis virus, rabies virus, mouse hepatitis virus, Borna disease virus, pseudorabies virus, and herpes simplex virus have all been demonstrated to enter the mouse CNS via olfactory neurons [34-40]. It is important to note that 2 of 62 mice tested had detectable virus within the brain without detectable virus in the nasal cavity (individual data not shown) suggesting that more than one route might be used to gain access to the CNS. We were surprised by the ability of the virus to infect intranasally, and found that the LD_50 _and ID_50 _were almost identical by either route by of inoculation. The kinetics of the development of clinical disease that occurred following intranasal administration of virus was similar for virus given IP or IN.

The finding that LACV is able to infect very efficiently via the nasal route has possible implications for the ecology of the virus. It is possible that infectious virus is present in water collections containing Aedes mosquito larvae, e.g., tree holes, since the virus has been shown to be transmitted from infected mosquitoes to larvae via eggs. A mammalian host that drinks the water would intake both fluid, which might contain free virus from lysed larvae, and infected larvae, either of which could initiate an infection in the mammalian host. Thus, exposure to such water could lead to an alternate route of infection for the natural hosts, i.e., the oral/nasal route in addition to the vector-bourne route. This hypothesis needs to be confirmed experimentally but remains an interesting possibility.

In the CNS of the weanling mouse, LACV infects predominantly neurons (some microglial cells are also infected) with spread in a rostral to caudal direction eventually reaching the lumbar spinal region. In both mice and humans, virus has been detected in the cerebral cortex, however infection appears more widespread in the mouse CNS with virus detection in the medulla oblongata, cerebellum, thalamus, olfactory bulb, and all regions of the spinal cord. The virus used in this study, LACV/human/1960, was isolated and passaged twice in C6/36 mosquito cells and was not previously adapted to growth in mouse neural tissue. One surprising difference between human and mouse infection was the detection of virus replication by IHC and the development of lesions in the mouse spinal cord [17].

Taken together, these data suggest that in weanling mice the virus first replicates in the periphery near the inoculation site. If the infection is not quickly controlled, the virus disseminates, most likely via blood, to the nasal turbinates. The detection of virus and lesions first in the rostral brain suggest the virus may utilize olfactory neurons to gain access to the CNS. The differences in findings between our study and previous work may be the result of differences in virus strain (mosquito vs. neurovirulent human isolates), mouse strain (outbred white vs. Swiss Webster), mouse age (suckling vs. weanling), inoculation route (subcutaneous vs. intraperitoneal) and dose (1000 TCID_50 _vs. 1 or 100 LD_50_). Although sequence data is not available for the strain used in the previous mouse pathogenesis work, it is known that the virus was a mosquito isolate and not directly linked to human disease [28].

In humans the majority of infections are asymptomatic, but children hospitalized with severe disease present with fever (86%), headache (83%), vomiting (70%), disorientation (42%), seizures (46%) and elevated peripheral white-cell counts (49%) [13]. Like the majority of human infections, rhesus monkeys in the current study experienced a subclinical infection without the development of systemic disease or neurologic symptoms. A much greater number of monkeys would probably need to be tested to detect neurologic symptoms after peripheral inoculation. Nevertheless, all monkeys were infected with LACV and developed neutralizing antibody responses, even after inoculation with as little as 10 PFU. Future work will include the intracerebral inoculation of rhesus monkeys to determine if LACV is neurovirulent in this species, but this will wait until vaccine candidates have been identified.

It is still unclear why so many human LACV infections remain asymptomatic. In our mouse model, infection with 1 LD_50 _of virus resulted in delayed CNS infection compared to mice receiving 100 LD_50_. Mice were able to control virus infection at doses at or below the LD_50, _and developed strong neutralizing antibody responses. The LACV ID_50 _for humans is unknown, but if human exposure is limited to a small dose, the virus may be effectively controlled by the immune system and CNS infection may be averted. If, like our mouse model, an individual is exposed to a greater dose of virus, virus growth may outpace control mechanisms leading to CNS infection. To further support the role of immune control affecting human disease outcome, it has been shown that individuals residing in endemic areas with major histocompatibility complex molecule B49 on CD8+ cytotoxic T lymphocytes (HLA-B49) had a greater relative risk of developing encephalitis after infection (relative risk 17.65, χ^2 ^= 7.3, P < 0.1). Infected individuals with HLA-DR5 had a lower relative risk of developing seizures (relative risk 0.22, χ^2 ^= 5.10, P < 0.025) [41].

## Conclusion

In weanling Swiss Webster mice, the LD_50 _and ID_50 _are similar, indicating that most infections lead to a lethal outcome at this age. LACV first replicates in tissues near the inoculation site, enters the blood stream, infects the nasal turbinates, and gains access to the CNS, presumably via olfactory neurons. This model will be useful to identify attenuated vaccine candidates that are deficient in the ability to disseminate from the site of inoculation, to replicate to high titers in the nasal turbinates, or to establish infection in mouse CNS. The CNS infection of mice appears more widespread than described for human infection, both in the brain and spinal cord. LACV is highly infectious both by the IP and IN routes suggesting that infection of natural mammalian hosts such as the chipmunk or squirrel might occur by the oral/intranasal route in addition to the bite of an infected mosquito. In rhesus monkeys, LACV is highly infectious with as little as 10 PFU resulting in the development of neutralizing antibodies, but clinical disease is not observed at any dose tested, suggesting that rhesus monkeys will be useful for studying the infectivity and immunogenicity of live attenuated virus vaccine candidates, but will be of limited usefulness for the evaluation of their level of attenuation.

## Methods

### Cells and viruses

C6/36 cells (*Aedes albopictus *mosquito larvae) were maintained in Earle's MEM supplemented with 10% fetal bovine serum (HyClone), 2 mM L-glutamine (Invitrogen), and 1 mM non-essential amino acids. Vero cells (African green monkey kidney) were maintained in OptiPRO™SFM medium (Invitrogen) supplemented with 4 mM L-glutamine (Invitrogen).

LACV/human/1960 was isolated from post-mortem brain tissue collected from a Minnesota patient hospitalized in La Crosse, Wisconsin and passaged two times in C6/36 cells. LACV/mosquito/1978 was isolated from mosquitoes collected in North Carolina and passaged once in mouse brain and three times in Vero cells. LACV/human/1978 was isolated from post-mortem brain tissue collected in Wisconsin and passaged once in mouse brain, twice in BHK-21 cells, and once in Vero cells. Biological clones were generated by terminal dilution in Vero cell cultures. Passage histories and complete genomic sequences for all stocks used in this paper have been previously published (EF485030-EF48538) [6].

### Determination of intranasal and intraperitoneal infectious dose of LACV

Weanling Swiss Webster mice (Taconic Farms, Germantown, NY) were inoculated with LACV/human/1960 diluted in L15 media (Invitrogen) intraperitoneally (100 μl volume) or intranasally (10 μl volume) while under isofluorane anesthesia. Mice were observed daily for 20 days for clinical disease including tremors, seizures, and limb paralysis. Moribund mice were promptly euthanized. Serum was collected 20 days after inoculation for determination of the presence and titer of neutralizing antibodies.

### LACV tissue distribution in mice

The replication of LACV virus was evaluated in 3-week-old weanling female Swiss Webster mice (Taconic Farms, Germantown, NY). All animal experiments were carried out in accordance with the regulations and guidelines of the National Institutes of Health. The Swiss Webster mice, were inoculated IP with 1 or 100 LD_50 _in a volume of 100 μl [6]. The tissues indicated in Table 1 were collected individually (3 mice per day for 6 days at each dose of virus), weighed, homogenized in L15 with SPG buffer (final concentration 218 mM sucrose, 6 mM L-glutamic acid, 3.8 mM dibasic potassium phosphate, pH 7.2). Homogenates were centrifuged for 10 minutes at 3000 rpm to remove cell debris and aliquots were frozen at -80°C until virus quantitation was performed. All mice were carefully observed twice daily for clinical disease including tremors and limb paralysis. Mice exhibiting clinical disease were promptly euthanized.

### Quantitation of virus in tissues

Monolayer cultures of Vero cells grown on 24-well plates were infected in duplicate with ten-fold serial dilutions of tissue homogenate, and the cells were overlayed with OptiMEM (Invitrogen) supplemented with 1% methylcellulose, 5% FBS, 2.5 μg/ml amphotericin B, and 20 μg/ml ciprofloxicin. Five days after infection the overlay was removed and cells were washed twice with PBS. The cells were fixed and stained for 10 minutes with crystal violet solution, virus plaques were enumerated, and tissue titers were expressed as mean log_10 _PFU/g tissue.

### Histopathology and immunohistochemical (IHC) staining

Weanling Swiss Webster mice were inoculated intraperitoneally with 100 LD_50 _of LACV/human/1960, and tissues were collected for six consecutive days for virus titration (n = 5) and pathology (n = 3) per day. Virus titrations were performed to confirm previous virus kinetics. Tissues collected for pathology were fixed in 10% neutral buffered formalin (NBF) for a minimum of 72 hours, embedded in paraffin and sections were prepared at 4–5 (μm). When bone was present in the tissue, as with muscle, nasal cavity, and spinal cord, tissues were decalcified using Immunocal (Decal Chemical Corp. Tallman, NY). Sections were stained with hematoxylin and eosin (H&E). A serial section was saved for immunohistochemical staining (see below).

For immunohistochemical analysis, fixed serial sections of mouse tissues were mounted onto slides and deparaffinized with xylene, rehydrated in a series of ethanol solutions (100%, 95%, 70%, 50%), and washed with deionized water. The sections were first treated with peroxidase blocking solution [2% H_2_O_2_(30%), 80% methanol, 18% dH_2_O] at room temperature for 5 minutes to quench endogenous peroxidases. Sections were pretreated with Pro K enzyme (Dako Corp., Carpinteria CA) at room temperature for 10 minutes and stained using the Mouse on Mouse Polymer detection system (Biocare Medical, Concord, CA). The primary antibody, mouse anti-La Crosse virus monoclonal antibody #18752 (QED Bioscience INC, San Diego, CA), recognized the G2 (Gn) glycoprotein and was used at a dilution of 1:200.

TUNEL Staining to detect apoptosis was preformed using the "DeadEnd™ Colorimetric TUNEL System" (Promega USA, Madison, WI). Sections were pretreated with Pro K enzyme (provided in the kit, diluted 1:500 with PBS). Strepavidin (also provided in the kit) was diluted at 1:500 in PBS.

To detect CD3+cells, slides were steam hydrated and pretreated with Diva Solution (Biocare Medical Concord, CA) for 20 minutes. The primary antibody, rabbit anti-human CD3, (A-0452, Dako Corporation, Carpentaria, CA) was used at a dilution of 1:300. The detection system was the Standard ABC kit (Vector Laboratories, Burlingame, CA), with 3,3'-diaminobenzidine (DAB) as the chromogen and a modified Harris hematoxylin counterstain.

To detect macrophage antigen 2 (MAC-2) expressing cells, slides were stream hydrated with citrate buffer for 20 minutes. The primary antibody, rat anti-Mac-2 (TIB-166, ATCC, Mannasas, VA) was used at a 1:200 dilution followed by a biotinylated goat-anti-rat IgG secondary antibody and developed with Streptavidin HRP (Biocare, Concord, CA).

### Inoculation of rhesus monkeys

Rhesus monkeys were inoculated with 10^5 ^PFU of biologically cloned human or mosquito LACV (LACV/human/1978-clone, LACV/mosquito/1978-clone). Each virus was inoculated intramuscularly (n = 4) or subcutaneously (n = 4). Blood samples were collected on days -7, 0, 2, 4, 6, 8, 10, 12, 14, 21, 28 post inoculation for blood chemistries, virus titration, and neutralizing antibody titration. Axillary or inguinal lymph nodes were surgically excised on days 4, 6, 8, and 12 post inoculation for viral titer or fixed in buffered formalin for histopathology and immunohistochemical analysis. Monkeys were observed daily for clinical disease. To determine LACV infectivity at low doses, rhesus monkeys (n = 4) were inoculated with 10^1 ^or 10^3 ^PFU LACV/mosquito/1978-clone subcutaneously. Blood samples were collected on days 0, 21, 28, and 42 post inoculation for neutralizing antibody titration.

### Neutralization assay

Neutralizing antibody in mouse and monkey serum was quantitated by a plaque reduction neutralization assay. Test sera were heat inactivated (56°C for 30 minutes) and serial 2-fold dilutions beginning at 1:5 or 1:10 were prepared in OptiMEM (Invitrogen) supplemented with 2% FBS, 50 μg/ml gentamicin, and 0.5% human albumin (Talecris Biotherapeutics, Inc., Research Triangle Park, NC). The homologous LACV was diluted to a final titer of 500 PFU/ml in the same diluent and 10% guinea pig complement (Cambrex Bioscience Walkersville, Inc., Walkersville, MD) was added to equal volumes of the serum dilutions and mixed well. Serum/virus mixture was incubated at 37°C for 30 minutes, added to confluent monolayers of Vero cells, and incubated for 1 hour to allow virus attachment. Cells were overlayed with 1% methylcellulose and incubated for 5 days at 37°C. After incubation, the overlay was removed, and the monolayers were washed twice with PBS and stained with crystal violet to allow for the enumeration of virus plaques. A 60% plaque-reduction neutralization titer was calculated.

## Competing interests

The author(s) declare that they have no competing interests.

## Authors' contributions

RSB performed animal studies, data analysis, and drafted the manuscript. CMC participated in mouse and monkey studies. CYF participated in the mouse studies. JMW oversaw pathology and provided all histopathologic and immunohistochemical data. BRM and SSW supervised the study and participated in its design and planning. All authors read and approved the final manuscript.

## References

[B1] Calisher CH (1994). Medically important arboviruses of the United States and Canada. Clin Microbiol Rev.

[B2] Rust RS, Thompson WH, Matthews CG, Beaty BJ, Chun RW (1999). La Crosse and other forms of California encephalitis. J Child Neurol.

[B3] Obijeski JF, Bishop DH, Palmer EL, Murphy FA (1976). Segmented genome and nucleocapsid of La Crosse virus. J Virol.

[B4] Gentsch JR, Rozhon EJ, Klimas RA, El Said LH, Shope RE, Bishop DH (1980). Evidence from recombinant bunyavirus studies that the M RNA gene products elicit neutralizing antibodies. Virology.

[B5] Schuh T, Schultz J, Moelling K, Pavlovic J (1999). DNA-based vaccine against La Crosse virus: protective immune response mediated by neutralizing antibodies and CD4+ T cells. Hum Gene Ther.

[B6] Bennett RS, Ton DR, Hanson CT, Murphy BR, Whitehead SS (2007). Genome sequence analysis of La Crosse virus and in vitro and in vivo phenotypes. Virol J.

[B7] Nasci RS, Moore CG, Biggerstaff BJ, Panella NA, Liu HQ, Karabatsos N, Davis BS, Brannon ES (2000). La Crosse encephalitis virus habitat associations in Nicholas County, West Virginia. J Med Entomol.

[B8] Gauld LW, Yuill TM, Hanson RP, Sinha SK (1975). Isolation of La Crosse virus (California encephalitis group) from the chipmunk (Tamias striatus), an amplifier host. Am J Trop Med Hyg.

[B9] Baldridge GD, Beaty BJ, Hewlett MJ (1989). Genomic stability of La Crosse virus during vertical and horizontal transmission. Arch Virol.

[B10] Gerhardt RR, Gottfried KL, Apperson CS, Davis BS, Erwin PC, Smith AB, Panella NA, Powell EE, Nasci RS (2001). First isolation of La Crosse virus from naturally infected Aedes albopictus. Emerg Infect Dis.

[B11] Cheng LL, Rodas JD, Schultz KT, Christensen BM, Yuill TM, Israel BA (1999). Potential for evolution of California serogroup bunyaviruses by genome reassortment in Aedes albopictus. Am J Trop Med Hyg.

[B12] Thompson WH, Kalfayan B, Anslow RO (1965). Isolation of California encephalitis group virus from a fata human illness. Am J Epidemiol.

[B13] McJunkin JE, de los Reyes EC, Irazuzta JE, Caceres MJ, Khan RR, Minnich LL, Fu KD, Lovett GD, Tsai T, Thompson A (2001). La Crosse encephalitis in children. N Engl J Med.

[B14] Jones TF, Craig AS, Nasci RS, Patterson LE, Erwin PC, Gerhardt RR, Ussery XT, Schaffner W (1999). Newly recognized focus of La Crosse encephalitis in Tennessee. Clin Infect Dis.

[B15] Huang C, Thompson WH, Karabatsos N, Grady L, Campbell WP (1997). Evidence that fatal human infections with La Crosse virus may be associated with a narrow range of genotypes. Virus Res.

[B16] McJunkin JE, Khan R, de los Reyes EC, Parsons DL, Minnich LL, Ashley RG, Tsai TF (1997). Treatment of severe La Crosse encephalitis with intravenous ribavirin following diagnosis by brain biopsy. Pediatrics.

[B17] Kalfayan B (1983). Pathology of La Crosse virus infection in humans. Prog Clin Biol Res.

[B18] Thompson WH, Kalfayan B, Anslow RO (1965). Isolation of California encepahalitis group virus from a fatal human illness. Am J Epidemiol.

[B19] Mack K, Salamon D, Dible M, Marcon M (1996). First Reported Recovery of LaCrosse Virus from CSF: a Case Study.. PanAmerican Society for Clinical Virology Clearwater Beach, Florida.

[B20] Chandler LJ, Borucki MK, Dobie DK, Wasieloski LP, Thompson WH, Gundersen CB, Case K, Beaty BJ (1998). Characterization of La Crosse virus RNA in autopsied central nervous system tissues. J Clin Microbiol.

[B21] Sokol DK, Kleiman MB, Garg BP (2001). LaCrosse viral encephalitis mimics herpes simplex viral encephalitis. Pediatr Neurol.

[B22] Wurtz R, Paleologos N (2000). La Crosse encephalitis presenting like herpes simplex encephalitis in an immunocompromised adult. Clin Infect Dis.

[B23] Balfour HH, Siem RA, Bauer H, Quie PG (1973). California arbovirus (La Crosse) infections. I. Clinical and laboratory findings in 66 children with meningoencephalitis. Pediatrics.

[B24] Utz JT, Apperson CS, MacCormack JN, Salyers M, Dietz EJ, McPherson JT (2003). Economic and social impacts of La Crosse encephalitis in western North Carolina. Am J Trop Med Hyg.

[B25] Amundson TE, Yuill TM (1981). Natural La Crosse virus infection in the red fox (Vulpes fulva), gray fox (urocyon cinereoargenteus), raccoon (Procyon lotor), and opossum (Didelphis virginiana). Am J Trop Med Hyg.

[B26] Amundson TE, Yuill TM, DeFoliart GR (1985). Experimental La Crosse virus infection of red fox (Vulpes fulva), raccoon (Procyon lotor), opossum (Didelphis virginiana), and woodchuck (Marmota monax). Am J Trop Med Hyg.

[B27] Ksiazek TG, Yuill TM (1977). Viremia and antibody response to La Crosse virus in sentinel gray squirrels (Sciuris carolinensis) and chipmunks Tamias striatus). Am J Trop Med Hyg.

[B28] Johnson RT (1983). Pathogenesis of La Crosse virus in mice. Prog Clin Biol Res.

[B29] Janssen R, Gonzalez-Scarano F, Nathanson N (1984). Mechanisms of bunyavirus virulence. Comparative pathogenesis of a virulent strain of La Crosse and an avirulent strain of Tahyna virus. Lab Invest.

[B30] Griot C, Gonzalez-Scarano F, Nathanson N (1993). Molecular determinants of the virulence and infectivity of California serogroup bunyaviruses. Annu Rev Microbiol.

[B31] Griot C, Pekosz A, Lukac D, Scherer SS, Stillmock K, Schmeidler D, Endres MJ, Gonzalez-Scarano F, Nathanson N (1993). Polygenic control of neuroinvasiveness in California serogroup bunyaviruses. J Virol.

[B32] Blakqori G, Delhaye S, Habjan M, Blair CD, Sanchez-Vargas I, Olson KE, Attarzadeh-Yazdi G, Fragkoudis R, Kohl A, Kalinke U, Weiss S, Michiels T, Staeheli P, Weber F (2007). La Crosse bunyavirus nonstructural protein NSs serves to suppress the type I interferon system of mammalian hosts. J Virol.

[B33] Gowen BB (2007). Animal models of highly pathogenic RNA viral infections:  Hemorrhagic fever viruses. Antiviral Research.

[B34] Lafay F, Coulon P, Astic L, Saucier D, Riche D, Holley A, Flamand A (1991). Spread of the CVS strain of rabies virus and of the avirulent mutant AvO1 along the olfactory pathways of the mouse after intranasal inoculation. Virology.

[B35] Lundh B, Kristensson K, Norrby E (1987). Selective infections of olfactory and respiratory epithelium by vesicular stomatitis and Sendai viruses. Neuropathol Appl Neurobiol.

[B36] Plakhov IV, Arlund EE, Aoki C, Reiss CS (1995). The earliest events in vesicular stomatitis virus infection of the murine olfactory neuroepithelium and entry of the central nervous system. Virology.

[B37] Huneycutt BS, Plakhov IV, Shusterman Z, Bartido SM, Huang A, Reiss CS, Aoki C (1994). Distribution of vesicular stomatitis virus proteins in the brains of BALB/c mice following intranasal inoculation: an immunohistochemical analysis. Brain Res.

[B38] Perlman S, Jacobsen G, Afifi A (1989). Spread of a neurotropic murine coronavirus into the CNS via the trigeminal and olfactory nerves. Virology.

[B39] Shankar V, Kao M, Hamir AN, Sheng H, Koprowski H, Dietzschold B (1992). Kinetics of virus spread and changes in levels of several cytokine mRNAs in the brain after intranasal infection of rats with Borna disease virus. J Virol.

[B40] Babic N, Mettenleiter TC, Ugolini G, Flamand A, Coulon P (1994). Propagation of pseudorabies virus in the nervous system of the mouse after intranasal inoculation. Virology.

[B41] Case KL, West RM, Smith MJ (1993). Histocompatibility antigens and La Crosse encephalitis. J Infect Dis.

